# Association between non-high-density lipoprotein cholesterol to high-density lipoprotein cholesterol ratio (NHHR) and hyperuricemia: evidence from the CHARLS study

**DOI:** 10.3389/fnut.2025.1552184

**Published:** 2025-04-25

**Authors:** Xin Hou, Zhenghao Zhu, Xinmin Chen, Yanhui Li, Guofeng Feng, Xiangjie Zhou, Zheng Gong, Yang Yang, Xiaohong Zhang

**Affiliations:** Department of Cardiology, The Third Affiliated Hospital of Anhui Medical University, Hefei, Anhui, China

**Keywords:** non-high-density lipoprotein cholesterol to high-density lipoprotein cholesterol ratio, hyperuricemia, CHARLS, non-HDL cholesterol, HDL cholesterol

## Abstract

**Background and aims:**

The non-high-density lipoprotein cholesterol to high-density lipoprotein cholesterol ratio (NHHR) is an innovative composite lipid measure. This study aims to examine the correlation between NHHR and hyperuricemia in the middle-aged and elderly demographic in China.

**Methods:**

This investigation comprised 4,639 individuals who were devoid of hyperuricemia at baseline in 2011, utilizing data from the China Health and Retirement Longitudinal Study (CHARLS). We utilized multivariable logistic regression, restricted cubic spline (RCS) analysis, and subgroup analysis to investigate the relationship between NHHR and hyperuricemia.

**Results:**

A total of 499 participants (10.76%) experienced hyperuricemia at the 4-year follow-up. The incidence of hyperuricemia was 176% higher for participants in the highest quartile of NHHR than for those in the lowest quartile (OR 2.76, 95% CI 2.10–3.62, *p* < 0.001). The risk of hyperuricemia was 64% higher in the highest quartile of NHHR than in the lowest quartile in a fully adjusted model (OR 1.64, 95% CI 1.16–2.31, *p* = 0.005). The risk of hyperuricemia and NHHR had a linearly positive connection, according to restricted cubic spline (RCS) analysis (*P* for non-linearity > 0.05). Subgroup analysis showed that among women, non-smokers, and those over 60, the relationship between NHHR and hyperuricemia was more significant.

**Conclusion:**

NHHR and hyperuricemia have a substantial linear positive connection, indicating that NHHR might be used as a tool for assessing hyperuricemia risk and offering valuable information for both prevention and therapy.

## Background

Hyperuricemia is a metabolic condition resulting from disruptions in purine metabolism, marked by increased plasma uric acid levels that are beyond normal thresholds. In recent years, the global prevalence of hyperuricemia has increased (1), progressively becoming a significant public health concern worldwide (1–3). The incidence of hyperuricemia in China is rising annually. In 2018–2019, the estimated prevalence of hyperuricemia among Chinese adults was 14.0%, with notable increasing trends seen between 2015–2016 and 2018–2019 (4). Hyperuricemia has emerged as the second most common metabolic condition following diabetes (5). Hyperuricemia is intricately associated with the pathogenesis of gout (6). Elevated uric acid levels serve as an early predictor and major cause of gout, while also representing important risk factors for hypertension, coronary heart disease, stroke, diabetes, chronic renal disease, and heightened mortality rates (7–11).

The cholesterol content of the majority of atherogenic lipoproteins, including low-density lipoprotein cholesterol (LDL-C), lipoprotein(a), intermediate-density lipoprotein, and remnants of very-low-density lipoprotein (VLDL) (12, 13), is represented by non-HDL-C, which is computed by deducting high-density lipoprotein cholesterol (HDL-C) from total cholesterol (14). On the other hand, HDL-C, which can help prevent atherosclerosis, is made up of the densest and smallest lipoprotein particles (15). A novel composite indicator for lipid evaluation, NHHR includes a variety of lipid particles that either promote or inhibit atherosclerosis. According to recent research, NHHR is very useful for determining the risk and prognosis of atherosclerosis and cardiovascular disease (16–18). However, it is also useful in assessing the risk of metabolic syndromes, including sarcopenia (19), diabetes mellitus (20), hypertension (21), diabetic kidney disease (22), and non-alcoholic fatty liver disease (23).

Hyperuricemia is a common metabolic condition in which uric acid acts as both an indicator of metabolic dysregulation and a facilitator of these disturbances (24). Dysregulation of lipid metabolism can modify the body’s metabolic processes and influence illness progression in individuals with hyperuricemia (25, 26). At present, only a few studies have evaluated the correlation between NHHR and the risk of hyperuricemia (27, 28). Nonetheless, these researches predominantly concentrated on populations in the United States, leaving the potential association ambiguous within the Chinese community. This study aims to investigate the potential correlation between NHHR and hyperuricemia utilizing the CHARLS database.

## Materials and methods

### Study population

This research constitutes a secondary analysis utilizing data from the China Health and Retirement Longitudinal Study (CHARLS). CHARLS^1^ is an extensive national cohort study designed to evaluate the economic, social, and health conditions of the population (29). The CHARLS cohort was formed by a multi-stage probability sampling method, with the baseline survey including 12,115 individuals aged 45 and above, drawn from 450 localities across 150 counties in 28 provinces between June 2011 and March 2012. The study design and cohort profile have been detailed in prior publications (29). The research obtained ethical clearance from the Biomedical Ethics Review Committee at Peking University, Beijing, China (IRB00001052-11015). All study participants furnished signed consent before enrollment. The dataset pertinent to this analysis is freely available on the CHARLS project website (29).

**Figure 1:**
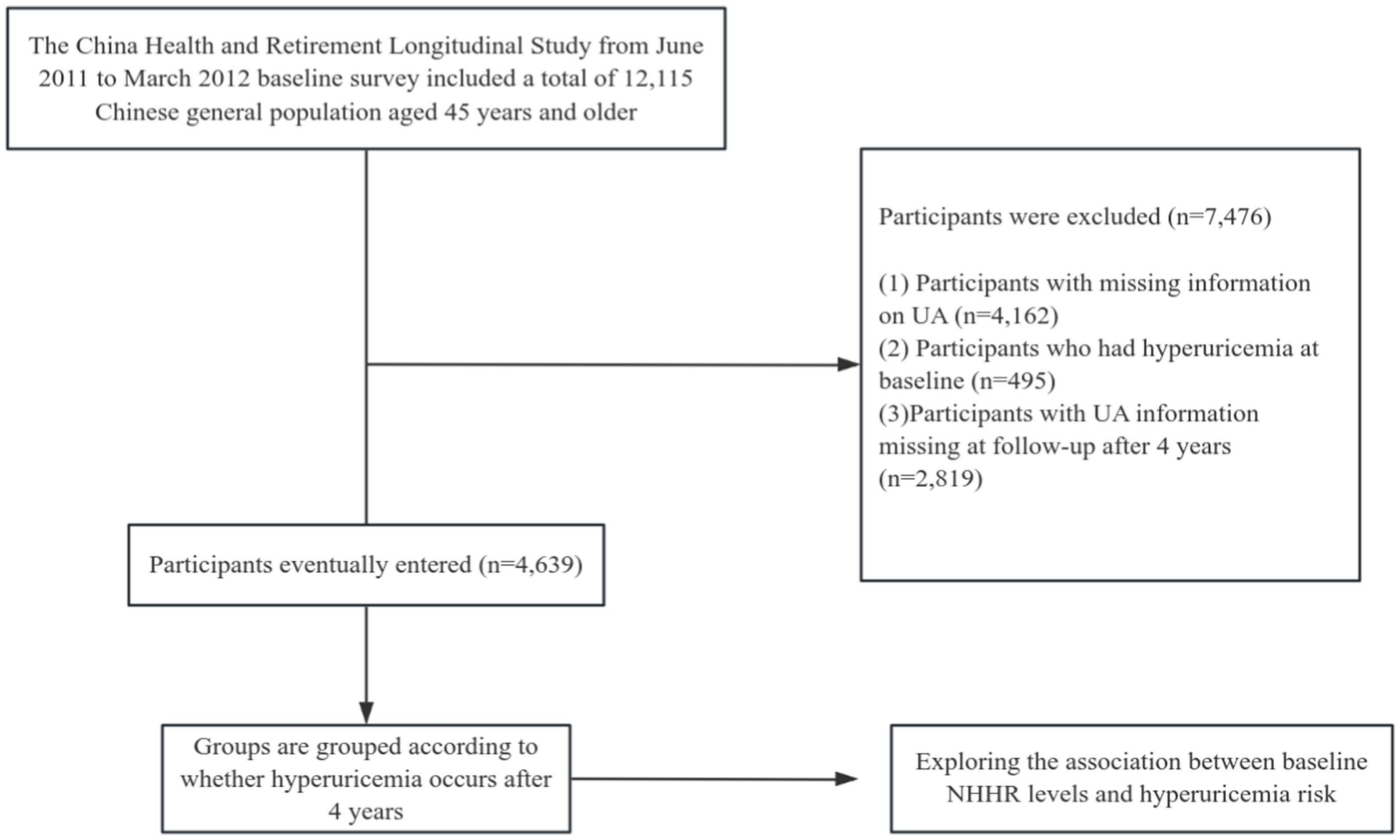
Flow chart of the study.

Our research employed data from the CHARLS survey carried out between 2011 and 2015. The initial survey (2011–2012) served as the baseline, and people devoid of hyperuricemia were incorporated into the study. The follow-up occurred in 2015–2016. Participants with incomplete serum uric acid data at baseline and those lacking serum uric acid data at follow-up were excluded from the trial (a comprehensive summary of the recruitment process is illustrated in Figure 1). A total of 4,639 patients were incorporated into this study and categorized according to the development of hyperuricemia after 4 years.

### Exposure and outcome definitions

Participants underwent overnight fasting before blood sample collection. The specimen was promptly frozen at −20 degrees Celsius and conveyed to the Chinese Center for Disease Control and Prevention in Beijing for analysis within a fortnight (30). Total cholesterol (TC) and HDL-C were quantified by skilled medical professionals utilizing enzymatic colorimetry. This study estimated non-HDL-C by subtracting HDL-C from total cholesterol (TC) (31, 32), and NHHR was derived as the ratio of non-HDL-C to HDL-C. Hyperuricemia is characterized by serum uric acid (SUA) concentrations of ≥7 mg/dL in males and ≥6 mg/dL in females (33).

### Covariates

Based on previous research and clinical expertise (34), this study considered various potential covariates, including age, gender, marital status, residence, education level, smoking status, drinking status, and self-reported chronic diseases [hypertension, diabetes mellitus (DM), heart disease]. Physical and chemical indicators included waist circumference (WC), body mass index [BMI = weight (kg)/height (m)^2^], systolic blood pressure (SBP), diastolic blood pressure (DBP), fasting plasma glucose (FPG), hemoglobin A1c (HbA1c), blood urea nitrogen (BUN), serum creatinine (Scr), cystatin C, triglycerides (TG), total cholesterol (TC), high-density lipoprotein cholesterol (HDL-C), low-density lipoprotein cholesterol (LDL-C), and the triglyceride-glucose (TyG) index (ln[FPG (mg/dL) × TG (mg/dL)/2]).

### Statistical analysis

Statistical analyses were performed utilizing SPSS software version 26.0 and R language software version 3.4.3. Statistical significance was established with a *p* value of less than 0.05 (two-tailed). This study experienced missing data, including age (3, 0.06%), marital status (3, 0.06%), residence (7, 0.15%), education level (9, 0.19%), smoking status (54, 1.16%), drinking status (3, 0.06%), hypertension (35, 0.75%), DM (58, 1.25%), heart disease (51, 1.10%), WC (609, 13.13%), BMI (637, 13.73%), SBP (621, 13.39%), DBP (621, 13.39%), FPG (1, 0.02%), HBA1c (35, 0.75%), Scr (3, 0.06%), cystatin C (1,148, 24.75%), TG (1, 0.02%), TC (1, 0.02%), LDL-C (8, 0.17%), and TyG index (1, 0.02%). The missing data analysis process assumed missing at random (MAR), and we created five imputed datasets, combining results using the multiple imputation method with chained equations and Markov Chain Monte Carlo (35, 36).

Participants were grouped based on whether hyperuricemia occurred at the 4-year follow-up, and baseline characteristics of individuals in the two groups were compared. Continuous variables are presented as mean ± standard deviation (SD), while categorical variables are described using percentages and frequencies. The differences between the two groups for categorical variables were compared using the *χ*^2^ test, and for continuous variables, the *t*-test was used.

Logistic regression analysis was utilized to investigate the relationship between NHHR and the occurrence of hyperuricemia. The variance inflation factor was further applied to detect multicollinearity in regression analyses. Four models were established sequentially: Model 1 did not adjust for any covariates, Model 2 adjusted for age (continuous) and gender, Model 3 adjusted for age, gender, marital status, residence, education level, smoking status, drinking status, self-reported chronic diseases (hypertension, DM, heart disease), WC, and BMI. Model 4 further adjusted for HbA1c, BUN, Scr, cystatin C, and the TyG index. To visualize the non-linear relationship between NHHR and hyperuricemia, we used restricted cubic spline (RCS) analysis, while considering the covariates from the aforementioned Model 4. The RCS model with three knots positioned at the 10th, 50th, and 90th percentiles was selected based on its minimal BIC value. Receiver operating characteristic (ROC) curve analysis was employed to evaluate the efficacy of NHHR, non-HDL-C, and TyG index in assessing the risk of hyperuricemia. Additionally, we conducted exploratory subgroup analyses to identify any statistically significant differences across subgroups, including age (<60 or ≥60 years), gender, marital status, residence, smoking status, alcohol consumption status, as well as history of hypertension, diabetes mellitus (DM), and heart disease.

## Results

### Baseline characteristics of participants

A total of 4,639 participants were analyzed and completed follow-up, comprising 2,101 males (45.29%) and 2,538 females (54.71%). The average age was 58.35 (9.10) years (Table 1). Compared to the non-hyperuricemia group, the hyperuricemia group was older and had a higher proportion of males, as well as more individuals with hypertension and heart disease (*p* < 0.05). Additionally, individuals in the hyperuricemia group exhibited elevated levels of systolic blood pressure (SBP), diastolic blood pressure (DBP), waist circumference (WC), blood glucose concentration, serum creatinine concentration, cystatin C concentration, triglyceride concentration, total cholesterol concentration, non-HDL cholesterol concentration, NHHR values, and the TyG index (*p* < 0.05). Conversely, a decrease in the proportion of married individuals and HDL cholesterol concentration was observed (*p* < 0.05).

**Table 1:** Baseline characteristics of participants grouped according to hyperuricemia.

Variables	Total (*n* = 4,639)	Non-hyperuricemia group (*n* = 4,140)	Hyperuricemia group (*n* = 499)	*P*
Age, years	58.35 ± 9.10	58.16 ± 9.00	59.92 ± 9.74	<0.001
Gender, *n* (%)				0.002
Female	2,538 (54.71)	2,298 (55.51)	240 (48.10)	
Male	2,101 (45.29)	1842 (44.49)	259 (51.90)	
Marriage status, *n* (%)				0.035
Not married	512 (11.04)	443 (10.70)	69 (13.83)	
Married	4,127 (88.96)	3,697 (89.30)	430 (86.17)	
Residence, *n* (%)				0.272
Urban	766 (16.51)	675 (16.30)	91 (18.24)	
Rural	3,873 (83.49)	3,465 (83.70)	408 (81.76)	
Education level, *n* (%)				0.755
Below primary school	2,219 (47.83)	1982 (47.87)	237 (47.49)	
Primary school	1,117 (24.08)	1,004 (24.25)	113 (22.65)	
Middle school	861 (18.56)	764 (18.45)	97 (19.44)	
High school or above	442 (9.53)	390 (9.42)	52 (10.42)	
Smoking status, *n* (%)				0.933
No	3,209 (69.17)	2,863 (69.15)	346 (69.34)	
Yes	1,430 (30.83)	1,277 (30.85)	153 (30.66)	
Drinking status, *n* (%)				0.081
No	3,144 (67.77)	2,823 (68.19)	321 (64.33)	
Yes	1,495 (32.23)	1,317 (31.81)	178 (35.67)	
Hypertension, *n* (%)	2043 (44.04)	1751 (42.29)	292 (58.52)	<0.001
DM, *n* (%)	636 (13.71)	559 (13.50)	77 (15.43)	0.237
Heart disease, *n* (%)	595 (12.83)	516 (12.46)	79 (15.83)	0.034
SBP, mmHg	127.76 ± 21.45	126.98 ± 21.05	134.23 ± 23.58	<0.001
DBP, mmHg	74.82 ± 12.38	74.41 ± 12.21	78.23 ± 13.30	<0.001
WC, cm	83.54 ± 13.09	83.20 ± 13.04	86.38 ± 13.18	<0.001
BMI, kg/m^2^	23.78 ± 13.06	23.71 ± 13.76	24.39 ± 3.68	0.270
FPG, mg/dL	108.12 ± 34.38	107.66 ± 33.16	111.99 ± 42.99	0.030
HBA1c, %	5.24 ± 0.81	5.23 ± 0.78	5.30 ± 0.98	0.123
BUN, mg/dL	15.70 ± 4.42	15.66 ± 4.43	16.04 ± 4.25	0.076
Scr, mg/dL	0.77 ± 0.18	0.76 ± 0.17	0.85 ± 0.20	<0.001
SUA, mg/dL	4.27 ± 1.04	4.16 ± 0.98	5.26 ± 0.96	<0.001
Cystatin C, mg/L	0.98 ± 0.23	0.98 ± 0.22	1.04 ± 0.27	<0.001
TG, mg/dL	132.74 ± 102.50	128.43 ± 95.51	168.52 ± 143.48	<0.001
TC, mg/dL	191.85 ± 38.00	191.26 ± 37.97	196.75 ± 37.95	0.002
Non-HDL-C, mg/dL	141.07 ± 37.92	139.95 ± 37.65	150.30 ± 38.86	<0.001
HDL-C, mg/dL	50.78 ± 15.06	51.31 ± 15.08	46.46 ± 14.13	<0.001
LDL-C, mg/dL	114.75 ± 34.42	114.67 ± 33.90	115.41 ± 38.49	0.681
TyG	8.67 ± 0.66	8.65 ± 0.64	8.90 ± 0.75	<0.001
NHHR	3.10 ± 1.68	3.03 ± 1.60	3.67 ± 2.18	<0.001

DM, diabetes mellitus; SBP, systolic blood pressure; DBP, diastolic blood pressure; WC, waist circumference; BMI, body mass index; FPG, fasting plasma glucose; HBA1c, hemoglobin A1c; BUN, blood urea nitrogen; Scr, serum creatinine; SUA, serum uric acid; TG, triglyceride; TC, total cholesterol; Non-HDL-C, non-high-density lipoprotein cholesterol; HDL-C, high-density lipoprotein cholesterol; LDL-C, low-density lipoproteins cholesterol; TyG, triglyceride glucose index; NHHR, non-high-density lipoprotein cholesterol to high-density lipoprotein cholesterol ratio.

### Association between NHHR and hyperuricemia

Table 2 indicates that 499 subjects (10.76%) exhibited hyperuricemia. Participants were categorized into four groups based on the quartiles of NHHR, with hyperuricemia incidence rates as follows: Quartile 1: 6.98%, Quartile 2: 8.54%, Quartile 3: 10.34%, and Quartile 4: 17.16%. To investigate the correlation between NHHR and the risk of hyperuricemia, we constructed four logistic regression models, as outlined in Table 2. In Model 1, participants in the highest NHHR quartile exhibited a 176% greater risk of hyperuricemia compared to those in the lowest quartile (OR 2.76, 95% CI 2.10–3.62, *p* < 0.001). In the fully adjusted model (Model 4), participants in the highest NHHR quartile exhibited a 64% greater risk of hyperuricemia compared to those in the lowest quartile (OR 1.64, 95% CI 1.16–2.31, *p* = 0.005).

**Table 2:** Correlation between NHHR and the risk of hyperuricemia in different models.

NHHR quartile	Events/subjects 499/4639	Model 1 OR (95%CI)	*P*	Model 2 OR (95%CI)	*P*	Model 3 OR (95%CI)	*P*	Model 4 OR (95%CI)	*P*
Q1	81/1160	Ref		Ref		Ref		Ref	
Q2	99/1159	1.24 (0.92–1.69)	0.161	1.29 (0.95–1.75)	0.104	1.24 (0.91–1.69)	0.177	1.07 (0.78–1.47)	0.695
Q3	120/1160	1.54 (1.15–2.06)	0.004	1.59 (1.19–2.14)	0.002	1.44 (1.07–1.95)	0.017	1.15 (0.84–1.59)	0.375
Q4	199/1160	2.76 (2.10–3.62)	<0.001	2.86 (2.18–3.77)	<0.001	2.51 (1.88–3.35)	<0.001	1.64 (1.16–2.31)	0.005

Model 1: Not adjusted.

Model 2: Adjusted for age and gender.

Model 3: Adjusted for age, gender, marital status, residence, education level, smoking status, drinking status, hypertension, DM, heart disease, WC, and BMI.

Model 4: Adjusted for age, gender, marital status, residence, education level, smoking status, drinking status, hypertension, DM, heart disease, WC, BMI, HBA1c, BUN, Scr, Cystatin C and TyG index.

**Figure 2:**
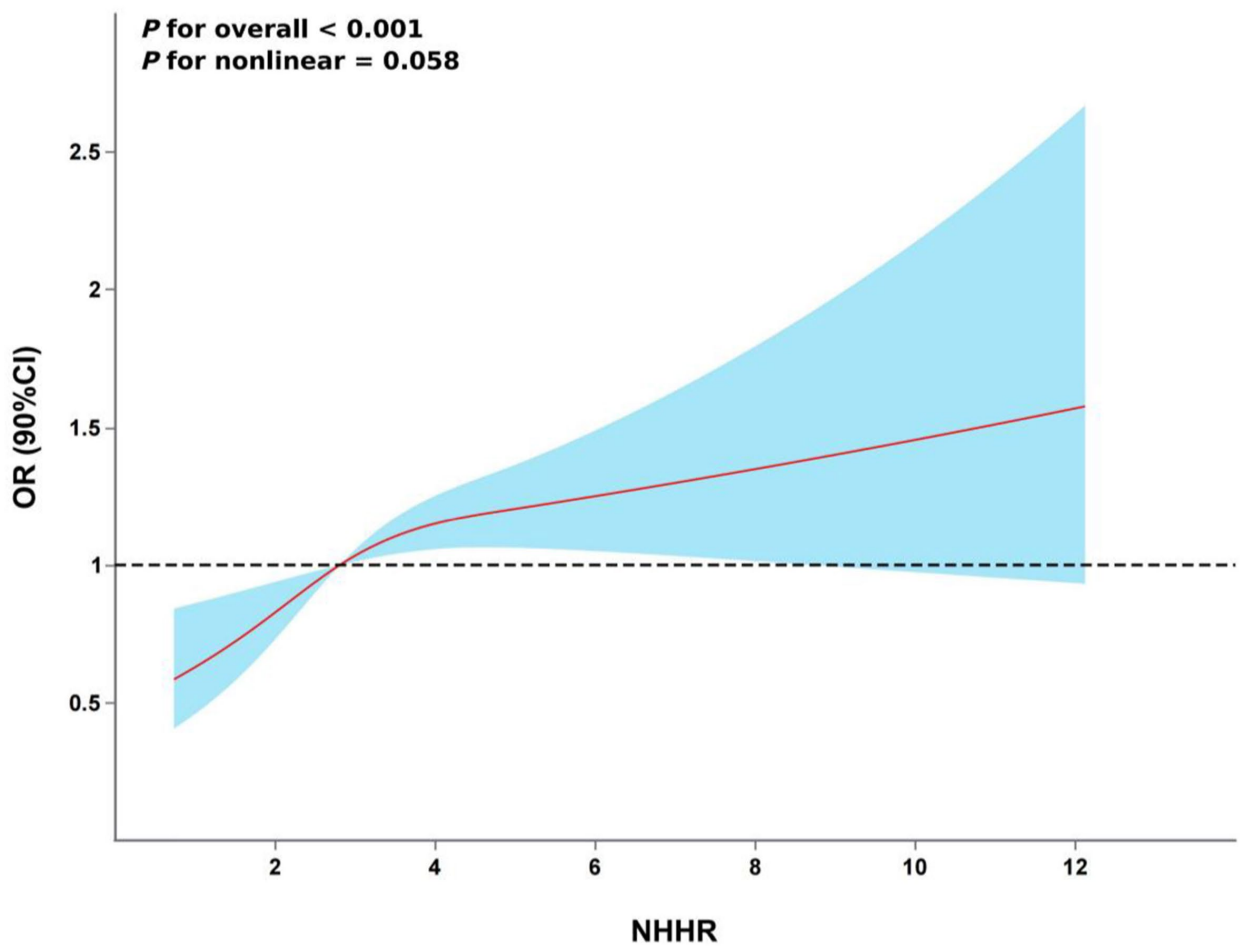
The association between NHHR and hyperuricemia risk. The model was adjusted for age, gender, marital status, residence, education level, smoking status, drinking status, hypertension, DM, heart disease, WC, BMI, HBA1c, BUN, Scr, Cystatin C and TyG index.

**Figure 3:**
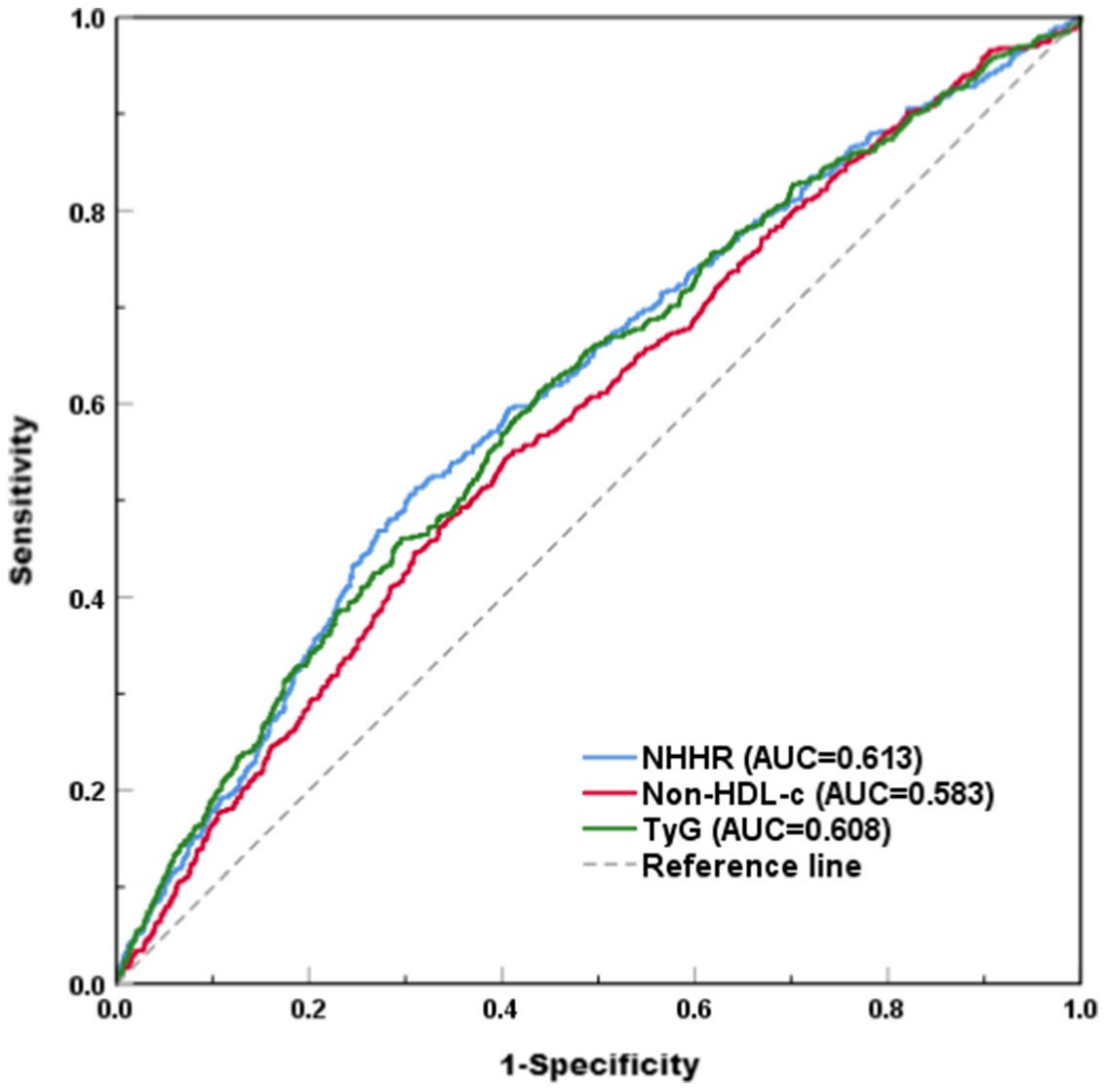
ROC curve.

The restricted cubic spline (RCS) analysis showed a linear relationship between NHHR and the risk of hyperuricemia after taking into account the same variables as in Model 4 (Figure 2, *P* for non-linearity > 0.05). There was no discernible threshold or saturation relationship; the risk of hyperuricemia rose by 7% for every standard deviation increase in NHHR (OR 1.07, 95% CI 1.00–1.15, *p* = 0.058). Additionally, according to the results of the ROC curve, the area under the curve (AUC) for the TyG index, non-HDL-C, and NHHR was 0.608, 0.583, and 0.613, respectively (Figure 3).

### Subgroup analysis

**Figure 4:**
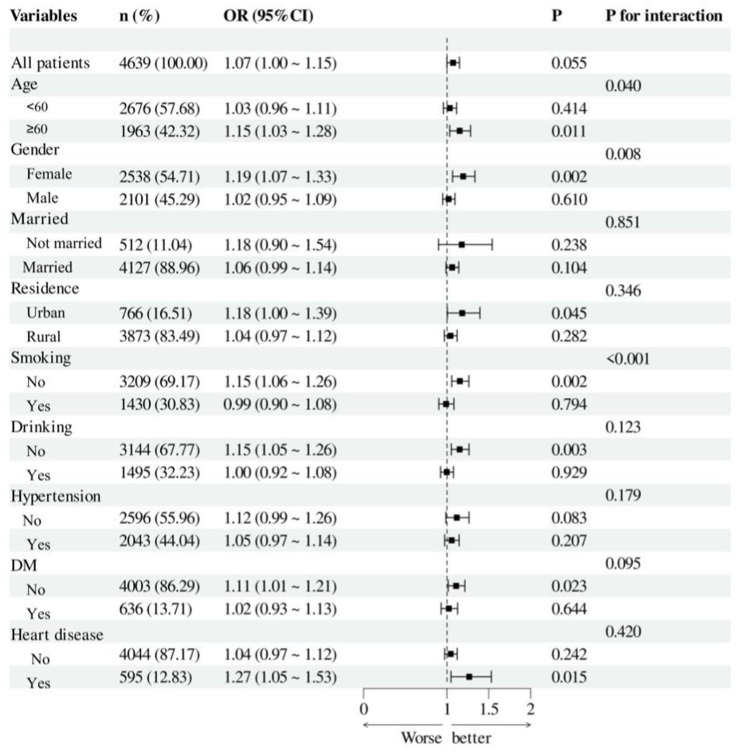
Forest plot of hyperuricemia risk according to different subgroups. The analysis with adjustment for age, gender, marital status, residence, education level, smoking status, drinking status, hypertension, DM, heart disease, WC, BMI, HBA1c, BUN, Scr, Cystatin C and TyG. In each case, the model is not adjusted for the stratification variable.

A exploratory subgroup analysis was performed to evaluate the consistency of the association between NHHR and hyperuricemia across various demographics (refer to Figure 4). We analyzed the interactions based on age (<60 years, >=60 years), gender, marital status, residence, smoking, and drinking status, as well as the history of hypertension, DM, and heart disease. The findings demonstrated that the correlation between NHHR and hyperuricemia in middle-aged and elderly adults in China was unaffected by marital status, residence, drinking status, or the history of hypertension, DM, and heart disease (interaction *p* > 0.05). We noted a significant interaction between NHHR and hyperuricemia across age, gender, and smoking status subgroups (*p* < 0.05), revealing a greater prevalence of hyperuricemia in adults aged over 60, females, and non-smokers.

## Discussion

This study utilized data from CHARLS 2011–2015, comprising 4,639 participants, to examine the potential correlation between NHHR and hyperuricemia. The findings demonstrated a favorable connection between NHHR and the incidence of hyperuricemia. The restricted cubic spline (RCS) analysis results indicated a linear positive connection between NHHR and hyperuricemia. The subgroup analysis indicated significant interactions among age, gender, and smoking status with NHHR levels, influencing the probability of hyperuricemia prevalence.

NHHR, a recently established lipid index, is largely utilized to evaluate atherosclerosis and is widely considered a significant marker of cardiovascular risk (16). It offers detailed information regarding both detrimental and beneficial lipid particles linked to atherosclerosis, potentially indicating the equilibrium between these two categories of lipoproteins (37). Research in metabolic diseases has demonstrated that NHHR has superior prediction powers for metabolic syndrome and insulin resistance (IR) (37, 38). Recent studies have demonstrated a strong correlation between NHHR and the prevalence of several metabolic disorders, including hypertension (21), DM (20), diabetic kidney disease (22), non-alcoholic fatty liver disease (23), sarcopenia (19), and hyperuricemia (28, 34). The association between uric acid and metabolic syndrome is intricate and reciprocal; increased uric acid levels are both a result of metabolic syndrome and a factor that can worsen its elements, including insulin resistance, obesity, and dyslipidemia. Conversely, insulin resistance enhances the renal reabsorption of uric acid, hence elevating uric acid concentrations (24). A growing body of research suggests that lipid metabolism plays a critical role in the emergence of hyperuricemia. Higher total cholesterol levels were found to be a risk factor for hyperuricemia, and a study carried out in northwest China revealed that dyslipidemia raises the prevalence of hyperuricemia (39). Higher triglyceride to HDL-C ratios were positively correlated with the prevalence of hyperuricemia, according to a four-year, large-scale cohort study conducted in China with 15,198 individuals by Liu et al. (40). Non-HDL-C was found to be the lipid index most substantially linked with hyperuricemia in prior studies conducted on middle-aged and older populations in China (32). Furthermore, research on additional markers of lipid metabolism, including residual cholesterol, the triglyceride-glucose index, and the triglyceride to HDL-C ratio, showed a linearly positive connection with hyperuricemia (41, 42). Increased NHHR may indicate a disruption in lipid metabolism in individuals, associated with the synthesis and elimination of uric acid. Nonetheless, research on the relationship between lipid ratios and the likelihood of developing hyperuricemia is sparse. Results from the National Health and Nutrition Examination Survey (NHANES) demonstrate that elevated NHHR levels correlate positively with hyperuricemia prevalence (28, 34). Our analysis utilizing the CHARLS revealed that, after adjusting for all relevant variables, individuals in the highest quartile of NHHR exhibited a 64% elevated risk of hyperuricemia relative to those in the lowest quartile (OR 1.64, 95% CI 1.16–2.31, *p* = 0.005). Subsequent restricted cubic spline (RCS) analysis revealed a linear positive connection between NHHR and the risk of developing hyperuricemia. Ultimately, subgroup analysis and interaction tests indicated that NHHR exhibited greater sensitivity in individuals aged over 60, females, and non-smokers. Prior cross-sectional research within the Chinese population revealed that the prevalence of hyperuricemia in males diminishes with advancing age, but females display a contrasting tendency (43). This may result from dietary practices, including the intake of high-purine meals, and lifestyle variations among middle-aged males, which may disguise the influence of NHHR, complicating the observation of this effect in subgroups. Prior research indicated a negative correlation between smoking and the prevalence of hyperuricemia among Chinese people (44, 45), aligning with our study findings.

The precise mechanism linking NHHR and hyperuricemia remains ambiguous. Potential causes encompass: (1) Increased lipid metabolism levels are strongly correlated with insulin resistance (46, 47). The lipid abnormalities associated with insulin resistance are directly linked to increased hepatic VLDL secretion, a central feature of the dyslipidemic profile characterized by elevated levels of small dense LDL particles and low HDL particle levels (48, 49). Studies indicate that HDL contributes to the maintenance of normoglycemia through both insulin-dependent and insulin-independent mechanisms (50, 51). Additionally, HDL suppresses stress-induced pancreatic *β*-cell death and may enhance glucose-stimulated insulin secretion, thereby contributing to its antidiabetic functions (52). Insulin resistance correlates with heightened expression of Urate transporter 1, facilitating uric acid reabsorption and impairing the activity of ATP-binding cassette sub-family G member 2, hence diminishing uric acid excretion (53), which results in raised serum uric acid concentrations. (2) Non-HDL-C constitutes a significant element of NHHR, encompassing LDL-C and residual cholesterol, both of which influence uric acid metabolism. Increased residual cholesterol amplifies the synthesis and consumption of free fatty acids, augments ATP catabolism, and elevates uric acid concentrations (54). It may also provoke insulin resistance, enhancing uric acid reabsorption in the proximal renal tubules (55). Oxidized LDL-C and lipid peroxidation cause disruptions in purine metabolism and elevate uric acid concentrations (56). (3) Reduced blood HDL-C levels might result in diminished glomerular filtration performance, consequently elevating serum uric acid levels. Reduced serum HDL-C levels are a significant contributor to atherosclerosis and a primary cause of renal artery stenosis and deteriorating glomerular filtration performance (57). Significant evidence indicates that low serum HDL-C levels correlate with the advancement of chronic renal disease (58). Additionally, HDL-C exhibits anti-inflammatory characteristics and can suppress endothelial inflammatory factors while downregulating the expression of the xanthine oxidase gene, thereby diminishing uric acid synthesis (59, 60). Reduced HDL-C levels may diminish antioxidant and anti-inflammatory properties (61). They can elicit inflammatory reactions and oxidative stress in the organism, enhance purine metabolism, and elevate uric acid synthesis (62, 63). Consequently, we can deduce that increased NHHR levels correlate with hyperuricemia.

This study has several advantages. This is the inaugural report of a linear positive connection between NHHR and hyperuricemia in the Chinese population. The data for this study are derived from the CHARLS database, which has a substantial sample size and is nationally representative. The effective management of potential confounding variables improves the reliability of the outcomes. Moreover, subgroup studies indicated that the association between NHHR and hyperuricemia is especially pronounced in those over 60 years of age, females, and non-smokers. It is important to emphasize that the subgroup analysis in this study carries the risk of multiple comparisons, which may lead to false-positive conclusions. Future research should validate these subgroup effects in independent cohorts.

However, this study is not without limitations. The cross-sectional approach hinders the discovery of definitive causal links, necessitating additional prospective investigations for confirmation. Although we accounted for numerous confounding variables, certain potential confounders remain unaccounted for. For instance, as demonstrated by Song et al. (64), a significant positive association exists between plasma aldosterone levels in hypertensive patients and the development of hyperuricemia and gout. Additional factors include the use of urate-lowering medications, lipid-lowering agents, and dietary habits characterized by excessive alcohol consumption and high-fat diets.

## Conclusion

In summary, NHHR, as a comprehensive lipid index, is closely associated with the occurrence of hyperuricemia. Therefore, monitoring NHHR in clinical practice may help to better assess patients’ metabolic status and provide important references for the prevention and treatment of hyperuricemia.

## Data Availability

Publicly available datasets were analyzed in this study. This data can be found at: http://www.isss.pku.edu.cn/cfps/.

## Footnotes

^1^ http://charls.pku.edu.cn/
